# Analysis of Clinical Course and Vaccination Influence on Serological Response in COVID-19 Convalescents

**DOI:** 10.1128/spectrum.02485-21

**Published:** 2022-04-04

**Authors:** Justyna Adamczuk, Piotr Czupryna, Justyna Dunaj-Małyszko, Ewelina Kruszewska, Sławomir Pancewicz, Karol Kamiński, Karol Borawski, Sambor Grygorczuk, Anna Moniuszko-Malinowska

**Affiliations:** a Department of Infectious Diseases and Neuroinfections, University Hospital in Białystok, Białystok, Podlaskie, Poland; b Department of Infectious Diseases and Neuroinfections, Medical University in Bialystok, Białystok, Podlaskie, Poland; c Department of Population Medicine and Lifestyle Diseases Prevention, Medical University of Bialystok, Bialystok, Podlaskie, Poland; d Department of Cardiology, University Hospital of Bialystok, Bialystok, Podlaskie, Poland; University of Manitoba

**Keywords:** COVID-19, anti-SARS-CoV-2 IgG antibodies, serology

## Abstract

Our goal was to assess the anti-SARS-CoV-2 antibodies presence in COVID-19 convalescents and assess the differences in anti-SARS-CoV-2 antibodies production regarding the disease severity, sex, vaccination, and assess the correlation between anti-SARS-CoV-2 antibodies production and inflammatory parameters. Three hundred twenty-two COVID-19 patients (282 hospitalized and 40 patients with oligosymptomatic COVID-19 isolated at homes) were included in the study. Blood was taken at 4 time points: during hospitalization, 1 month, 3 months, and 6 months. Detection of SARS-CoV-2 antibodies was performed with LIAISON SARS-CoV-2 S1/S2 IgG tests (DiaSorin, Italy). Clinical and laboratory parameters were compared. Significant differences between higher anti-SARS-CoV-2 antibodies titer in symptomatic patients 3 months after infection (III sample) and significantly higher ratio II/I in symptomatic patients were observed. Subgroup analysis based on sex showed differences only in laboratory tests, not in serological. Analysis of the results of serological tests showed significant differences in ratio IV/I and a significant increase in antibodies level after vaccination. The most significant rise was observed between the 3rd and 6th month when the patients received a vaccination. Immunological response after COVID-19 infection lasted over 6 months in all patients, although antibodies titers were significantly higher in patients with a history of severe COVID-19 and vaccinated patients. Immunological response after COVID-19 infection did not depend on sex. There was a significant correlation between anti-SARS-CoV-2 antibodies production and the degree of inflammation in the acute phase of the disease (inflammatory parameters in blood and severity of lung affection in CT).

**IMPORTANCE** The results of our study confirm the knowledge on immune response in the Polish population and add new information regarding correlations with the severity of the disease. The data in the literature concerning the correlation between antibodies response and sex are ambiguous, and we did not observe differences between antibodies production and gender, which also adds new information.

## INTRODUCTION

COVID-19 pandemic is recently the most important topic as far as infectious diseases are concerned. Since March 2020, SARS-CoV-2 has been responsible for 428 M infections and 5.91 M deaths (1). The most effective way of COVID-19 prevention is vaccination. One of the potential problems with managing COVID-19 patients is the lack of data concerning neutralizing antibodies dynamics in the population, which could provide useful information for future changes in vaccination recommendations.

Most patients infected with SARS-CoV-2 are asymptomatic or mildly symptomatic, while in minority the disease will take a severe course with acute respiratory distress syndrome (ARDS), extensive inflammation, and the so-called cytokine storm. Symptomatic patients with severe course require hospitalization (2).

The antibodies are detectable as soon as 6 days after PCR confirmation of infection. The antibodies mainly target the spike (S) and nucleocapsid proteins (NCP) of the SARS-CoV-2 (3). The S protein is the principal determinant of protective immunity and cross-species transmission in SARS-CoV-2 and monoclonal antibodies against the S protein could neutralize viral infectivity (4, 5). On this basis, Walls et al. (6) hypothesized that exposure to SARS-CoV-2 could elicit mutually cross-reactive, potentially neutralizing antibodies. The N Protein is located in the core of the virus. The effect of high titers of IgG against N-protein on clinical outcomes of SARS-CoV-2 disease has not been described.

COVID-19 convalescents are an important group as we still do not know which factors influence the immunological response of the host and whether these patients are even partially immune against reinfection.

Therefore, the current study aimed to assess the anti-SARS-CoV-2 antibodies presence in COVID-19 convalescents.

Our detailed aims were:

(i) To assess the differences in anti-SARS-CoV-2 antibodies production based on the disease severity.

(ii) To assess the differences in anti-SARS-CoV-2 antibodies production based on the vaccination status.

(iii) To assess the time trend in anti-SARS-CoV-2 antibodies production.

(iv) To assess the correlation between anti-SARS-CoV-2 antibodies production and inflammatory parameters.

## RESULTS

### General results.

Laboratory parameters and anti-SARS-CoV-2 antibodies levels of all patients included in the study at admission are presented in Table 1.

**TABLE 1:** Laboratory parameters and anti-SARS-CoV-2 antibodies levels of all patients included in the study at admission

Laboratory parameters	Mean	SD	Median	Min	Max
Age	59.1	15.72	60	19	94
Laboratory tests					
CRP (mg/L)	73	70.13	50	0.30	328.4
Procalcitonin (ng/mL)	0.2	0.63	0.1	0.01	6.6
WBC (1000/μL)	6.4	3.6	5.6	1.4	40.6
PLT (1000/μL)	205.8	897.02	191	42	933
IL-6 (pg/mL)	69.4	97.59	43.5	1.50	1170.5
d-dimer (ng/mL)	2539.8	10473.33	820.0	106	102965
ALT (IU/L)	41.3	36.03	30.0	5.00	305
Serology					
Anti-SARS-CoV-2 antibodies I	78.4	83.4	63.4	1.00	535
Anti-SARS-CoV-2 antibodies II	174.8	192.28	126	1.00	1460
Anti-SARS-CoV-2 antibodies III	289.3	655.62	130.5	1.00	4970
Anti-SARS-CoV-2 antibodies IV	1827.7	4096.28	390	0.00	20000
Ratio II/I	18.5	48.07	1.9	0.00	277
Ratio III/I	20.6	69.52	1.6	0.00	400
Ratio IV/I	13.2	28.78	1.0	0.00	128.2

### Comparison of the results of serological tests based on the course of the disease.

**FIG 1:**
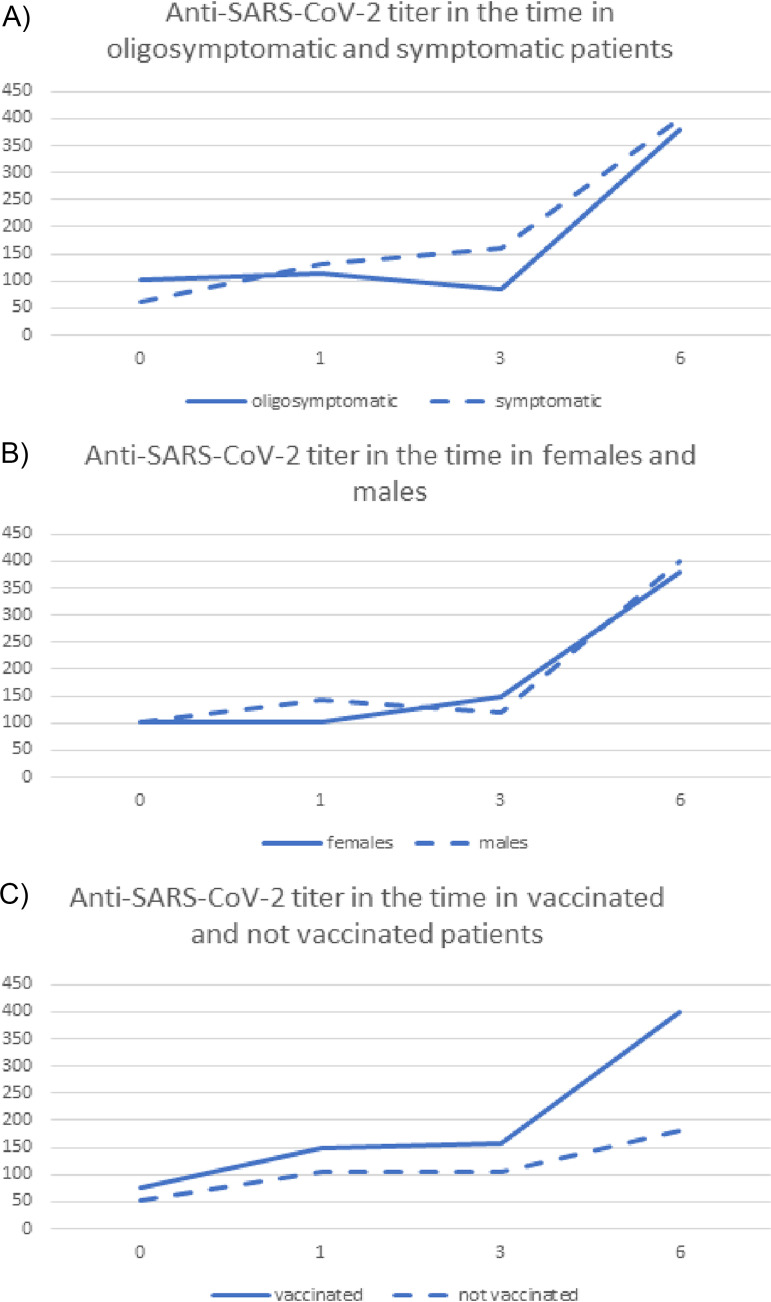
(A) The titer of anti-SARS-CoV-2 IgG antibodies in people after oligosymptomatic versus symptomatic COVID-19 infection. (B) The titer of anti-SARS-CoV-2 IgG antibodies in people after COVID-19 infection in relation to sex. (C) The titer of anti-SARS-CoV-2 IgG antibodies in people after COVID-19 infection in vaccinated and unvaccinated patients against SARS-CoV-2.

A comparison of the results of serological tests based on the course of the disease is presented in Table 2 and Fig. 1A.

**TABLE 2:** Comparison of the serological tests based on the course of the disease

Laboratory parameters	Symptomatic course *n* = 282					Oligosymptomatic course *n* = 40					*P* value
	Mean	SD	Median	Min	Max	Mean	SD	Median	Min	Max	
Age	59.4	15.95	60	19	94	55.83	13.173	57.5	38	81	NS
Serology											
Anti-SARS-CoV-2 antibodies I	70.1	63.22	60.4	1.0	307	121.389	139.87	101.25	1.0	535	NS
Anti-SARS-CoV-2 antibodies II	182.3	203.88	131.0	1.0	1460	157.003	157.45	114	1.0	712	NS
Anti-SARS-CoV-2 antibodies III	365	767.93	162.0	1.0	4970	114.985	117.83	83.5	1.000	400	0.002
Anti-SARS-CoV-2 antibodies IV	1784.5	3892.97	400	36.5	20000	2034.253	4718.07	380	0.0008	18500	NS
Ratio II/I	22.1	52.6	2.0	0.02	277.0	2.979	5.8	1.3	0.0	24.5	0.03
Ratio III/I	23.2	74.94	1.7	0.0	400.0	5.59	12.19	1.5697	0.0	38.0	NS
Ratio III/II	8.0	42.35	0.8	0.0	337.0	1.126	0.73	1.0099	0.0575	3.6	NS
Ratio IV/I	12.8	29.09	1.0	0.00	128.2	21.41	28.86	21.4095	1.0008	41.82	NS
Ratio IV/II	192.8	977.54	2.0	0.19	5180.0	20.51	34.27	6.4771	0.04	92.65	NS
Ratio IV/III	6.4	11.26	1.6	0.32	50.0	11.779	25.57	1.6965	0.591	79.06	NS

Analysis of the results of serological tests, depending on the course of the disease, showed significant differences between higher anti-SARS-CoV-2 antibodies titer in symptomatic patients 3 months after infection and significantly higher ratio II/I.

### Comparison of the results of serological tests based on sex.

Analysis of the subgroups depending on sex showed differences only in laboratory tests, but not in serological test results (Table 3, Fig. 1B).

**TABLE 3:** Comparison of the results of serological tests based on sex

Laboratory parameters	Female *n* = 165					Male *n* = 157					*P* value
	Mean	SD	Median	Min	Max	Mean	SD	Median	Min	Max	
Age	61.4	15.94	62	19	94	56.6	15.1	56	22	92	0.05
Biochemical tests											
CRP (mg/liter)	62.6	68.11	35.3	0.3	321.5	83.8	70.8	60.8	0.65	328.4	0.05
Procalcytonin (ng/mL)	0.2	0.6	0.1	0.03	4.2	0.2	0.7	0.1	0.01	6.6	0.05
WBC (1/μL)	5891.3	2791.54	5080	1400	17360	6850.2	4153.2	6110	2470	40570	0.05
PLT (1/μL)	203153.8	74098.18	190000	42000	463000	208630.4	103628.8	191000	43000	933000	NS
IL-6 (pg/mL)	58.2	59.99	38.3	1.5	303.5	80	122.6	50	1.5	1170.5	NS
d-dimer (ng/mL)	2385.4	9695.09	812	106	93239	2699.9	11258.4	845.5	138	102965	NS
ALT (IU/liter)	37.9	41.72	25	7	305	44.8	28.7	37	5	142	0.05
Serology											
Anti-SARS-CoV-2 antibodies I	104.9	89.55	101	13.1	535	97.1	54.7	101	13.5	307	NS
Anti-SARS-CoV-2 antibodies II	196.4	238.88	101	21.5	1460	171.5	123	142.5	10	580	NS
Anti-SARS-CoV-2 antibodies III	258.9	416.98	150	16.8	2880	339.8	836	121	29.1	4970	NS
Anti-SARS-CoV-2 antibodies IV	2278.6	5103.48	380	16	20000	1257.1	1924.3	400	46.4	7590	NS
Ratio II/I	3	3.08	1.9	0.34	12.1	2.2	1.6	1.5	0.15	6.7	NS
Ratio III/I	3.9	3.34	2.4	1.01	12.9	7.9	24.9	2	0.76	113.2	NS
Ratio IV/I	30.7	40.73	8.3	0.59	128.2	18.9	30.6	3.3	1.24	85.2	NS

### Comparison of the results of serological tests based on the vaccination history.

A comparison of the results of serological tests based on the vaccination history is presented in Table 4. All patients who were vaccinated were vaccinated between third and fourth sample taking. The median time between vaccination and examination was 28 days.

**TABLE 4:** Comparison of the results of serological tests based on the vaccination history

Laboratory parameters	Vaccinated patients *n* = 93					Nonvaccinated patients *n* = 229					*P* value
	Mean	SD	Median	Min	Max	Mean	SD	Median	Min	Max	
Age	58.1	12.44	60.5	27	84	59.5	16.92	60	19	94	NS
Biochemical tests											
CRP (mg/liter)	68.1	73.69	40.6	0.49	287.4	74.6	69.08	51.4	0.3	328.4	NS
Procalcytonin (ng/mL)	0.2	0.48	0.1	0.05	2.8	0.2	0.67	0.1	0.01	6.6	NS
WBC (1/μL)	6133.1	2391.94	5370	2550	13250	6434	3846.88	5670	1400	40570	NS
PLT (1/μL)	212373.1	93296.99	197000	42000	480000	203799.1	88671.95	187000	43000	933000	NS
IL-6 (pg/mL)	60.7	61.86	32.5	2.9	267.1	71.4	104.21	45.4	1.5	1170.5	NS
d-dimer (ng/mL)	1959.6	7796.02	747	106	63564	2721.1	11189.49	823	138	102965	NS
ALT (IU/liter)	43	35.71	34	11	241	40.7	36.21	30	5	305	NS
Serology											
Anti-SARS-CoV-2 antibodies I	87.4	96.21	75.5	1.0	535	67.7	61.12	54.0	1	207	NS
Anti-SARS-CoV-2 antibodies II	193.7	211.65	149.5	1.0	1460	146.3	154.22	106.5	1.0	726	NS
Anti-SARS-CoV-2 antibodies III	366.4	802.97	157	1.0	4970	158.0	169.99	105	1.0	706	NS
Anti-SARS-CoV-2 antibodies IV	2223.8	4598.68	400	1.0	20000	621.5	1191.34	181.5	19.8	3910	NS
Ratio II/I	19.6	55.2	1.7	1000	277	16.9	35.6	1.9	0.15	168	NS
Ratio III/I	28.6	84.38	2	0.0	400	5.3	15.53	1.1	0.0	71.7	NS
Ratio IV/I	17.2	33.81	1.3	0.0	128.2	5.5	12.91	0.8	0.0	41.8	0.04

Analysis of the results of serological tests when dividing the groups according to the vaccination history showed significant differences in ratio IV/I. Ninety-three patients were vaccinated and analysis of anti-SARS-CoV-2 antibodies with Wilcoxon test showed a significant increase in the antibodies level in this subgroup (Fig. 1C). The most significant rise was observed between the 3rd and 6th month (III and IV sample) when most people received a vaccination. The rising trend in antibody production between the 3rd and 6th month was mirrored in the nonvaccinated group, although the increase was nonsignificant.

### Analysis of correlation of anti-SARS-Cov-2 antibodies titer and selected laboratory and radiological parameters.

Percentage of lungs affected by the COVID-19 in chest CT correlated with anti-SARS-CoV-2 antibodies II (*R* = 0.3, *P* < 0.05). C reactive protein (CRP) before treatment correlated with anti-SARS-CoV-2 antibodies I (*R* = 0.22, *P* < 0.05), anti-SARS-CoV-2 antibodies II (*R* = 0.37, *P* < 0.05) and anti-SARS-CoV-2 antibodies III (*R* = 0.39, *P* < 0.05). Procalcitonin correlated with anti-SARS-CoV-2 antibodies II (*R* = 0.22, *P* < 0.05). IL-6 before treatment correlated with anti-SARS-CoV-2 antibodies II (*R* = 0.25). d-dimer before treatment correlated with anti-SARS-CoV-2 antibodies II (*R* = 0.41) and anti-SARS-CoV-2 antibodies III (*R* = 0.25). Alanine transaminase (ALT) before treatment correlated with anti-SARS-CoV-2 antibodies II (*R* = 0.39).

## DISCUSSION

Our study supports the observation of other authors regarding the serological response after SARS-CoV-2 infection. It showed that all examined convalescents produced antibodies against SARS-CoV-2.

Infection with SARS-CoV-2 leads to an antibody response, even in completely asymptomatic patients. However, the initial immune response is not as strong as in patients with more severe diseases. Choe et al. (7) evaluated the antibody responses of 58 persons in South Korea and found out that, after 8 months in asymptomatic or mildly symptomatic patients, SARS-CoV-2 infection antibodies were still detected (anti-N pan-Ig in 91.4%, anti-N IgG in 25.9%, anti-S IgG in 86.2%, and anti-S1 IgG in 69.0%) (7).

In our study, patients with severe COVID-19 had a stronger immunological response which is indicated by a higher II/I ratio and higher median antibodies titer 3 months postinfection. This is in accordance with Trogakos et al. (8), who observed that severe COVID-19 (versus moderate disease) triggers an earlier and more intense immune response in hospitalized patients; in all cases, however, antibody titers remain at high levels in COVID-19 recovered patients. Similar to Choe et al. (7), we observed that even in oligosymptomatic patients the antibodies titer did not significantly decrease after 6 months.

We observed a correlation of serological response 1 month after infection with the intensity of inflammatory lesions in chest CT (presented as percentage of lungs affected) and all-important blood inflammatory markers (procalcitonin, CRP, d-dimer, IL-6), which additionally confirms the influence of the disease severity on humoral immunological response.

Terpos et al. (9) recently reported that female sex and young age predisposed to more intense immunological response after COVID-19. Nonetheless, we did not observe this correlation in our study.

Hartley et al. proved that COVID-19 patients rapidly generate B cell memory to both spike and nucleocapsid antigens following SARS-CoV-2 infection. It has also been reported that antibody levels to SARS-CoV-2 decrease over time and this decrease reflects a contraction phase of the immune response (10).

The efficacy of vaccination has already been demonstrated in many studies (11–16), and vaccination likely offers more protection than natural infection. The vaccine is also likely to be sufficient to trigger secondary boosting immune responses in COVID-19 recovered patients being positive for anti-S-RBD (receptor binding domain) IgGs/Nabs (8). In our vaccinated patients, there was a strong rise in antibodies titer between the third and fourth sample, demonstrating the boosting effect of the vaccine, while in the nonvaccinated group the increase was much lower.

The limitation of the study was a small sample of patients (especially with oligosymptomatic COVID-19). Another limitation of our study was the fact, that the patients enrolled in the study were middle-aged or senior, and very little data are available in young asymptomatic or mildly symptomatic subjects. However, the hospitalized patients are usually older than asymptomatic or oligosymptomatic patients. As our study concentrated mostly on hospitalized patients, the gathered data reflect the results in these age groups. The vaccination process in the examined group was also not homogenous and patients were vaccinated at different time points (between 3rd and 6th month) which could have affected the results. Moreover, it would be interesting to know the influence of the SARS-CoV-2 variant on the intensity of the immune response, but unfortunately, we have not obtained these data, which we consider another limitation of the study. However, we used data from the integrated real-time (RT) monitoring process for variants and SARS-CoV-2 mutation Map “RT-COVAR” for retrospective analysis of SARS-CoV-2 variants affecting the Polish population in the analyzed time frame (17). On the national level, the virtual map “RT-COVAR” is used daily by the Ministry of Health and the National Institute of Hygiene to create the country's epidemiological policy. The data on the diversity of the SARS-CoV-2 virus genome in Poland are used by global databases such as GISAID, which are then used to create special recommendations by institutions like the FDA or WHO. When we analyzed our time frame, we knew that we dealt with wild-type variants and Alpha and Delta variants. Further studies in this area would bring new knowledge about serological responses depending on variants of the virus.

Another limitation worth discussing is the potential cross-reactivity of the test used. The cross-reactivity study for the LIAISON SARS-CoV-2 S1/S2 IgG assay was designed to evaluate potential interference from antibodies to other viruses that may cause symptoms similar to SARS-CoV-2 infection, other organisms that may cause infectious diseases, as well as from other conditions that may result from atypical immune system activity. *In vitro*, three specimens out of 165 assessed resulted positive with the LIAISON SARS-CoV-2 S1/S2 IgG assay and they were: anti-HBV, anti-influenza A, rheumatoid factor. The observed specificity for potentially cross-reactive specimens is comparable to that of open populations (18).

Our conclusions include:

(i) Immunological response after COVID-19 infection lasts over 6 months in all patients although antibodies titers are significantly higher in patients with a history of severe COVID-19 and vaccinated patients.

(ii) Immunological response after COVID-19 infection does not depend on sex.

(iii) There is a significant correlation between anti-SARS-CoV-2 antibodies production and the degree of inflammation in the acute phase of the disease (inflammatory parameters in blood and severity of lung affection in CT).

## MATERIALS AND METHODS

### Material and patient group.

Three hundred twenty two patients (282 hospitalized and 40 patients with oligosymptomatic COVID-19 isolated at home) in the mean age 59.1 ± 15.72 years old of both sexes (165 females and 157 males) with a history of SARS-CoV-2 infection were included in the study. The diagnosis of SARS-CoV-2 infection was confirmed by reverse transcription-PCR (RT-PCR) testing by the CFX96 Real-Time System (Bio-Rad) from nasopharyngeal or oropharyngeal swabs. Patients had no previous history of SARS-CoV-2 infection.

### Serological analyses.

The blood was taken at 4 time points from 29.02.2020 until 21.05.2021: during hospitalization (I sample), 1 month (II sample), 3 months (III sample), 6 months (IV sample) after the hospitalization. The ratios of II/I, III/I, IV/I, III/II, IV/II, IV/III were calculated.

Ninety-three patients were vaccinated (various vaccines) between third and fourth sample taking. In oligosymptomatic patients, only serology samples were obtained, while laboratory or radiological examinations were not performed.

Detection of SARS-CoV-2 antibodies was performed with LIAISON SARS-CoV-2 S1/S2 IgG tests (DiaSorin, Italy). This is a quantitative assay for the detection of IgG antibodies against S1/S2 antigens of SARS-CoV-2. The assessment was performed with a fully automated solution on LIAISON XL enabling the detection of neutralizing antibodies: 94.4% positive agreement to Plaque Reduction Neutralization Test (PRNT90).

Clinical and laboratory parameters were analyzed. Laboratory parameters, such as white blood cell (WBC), neutrophils, lymphocytes, platelet (PLT), d-dimer, procalcitonin, CRP, and PLT were determined before and after treatment. All patients were asked about vaccination history (type of vaccine, number of doses, time of vaccination).

The study was approved by the Ethical Committee of Medical University of Bialystok, Poland. All patients signed a consent to participate in the study.

### Statistical analysis.

The statistical analysis was performed using the Statistica 13.0 program. Data were presented as means and standard deviations or medians, minimum and maximum, as appropriate. The normal distribution was evaluated by the Shapiro-Wilk test. In statistical analysis, the Mann-Whitney U test or paired Wilcoxon singed-rank test were used as appropriate.

Correlations were measured with Spearman’s Rank test.

A probability level *P* < 0.05 was considered statistically significant.

### Data availability.

The data that support the findings will be available on request under the corresponding author’s e-mail: annamoniuszko@op.pl.
